# Association between Systolic Blood Pressure and Diabetic Retinopathy in Both Hypertensive and Normotensive Patients with Type 2 Diabetes: Risk Factors and Healthcare Implications

**DOI:** 10.3390/healthcare9050580

**Published:** 2021-05-13

**Authors:** Yu-Ting Li, Yi Wang, Xiu-Jing Hu, Jia-Heng Chen, Yun-Yi Li, Qi-Ya Zhong, Hui Cheng, Bedru H. Mohammed, Xiao-Ling Liang, Jose Hernandez, Wen-Yong Huang, Harry H. X. Wang

**Affiliations:** 1 State Key Laboratory of Ophthalmology, Zhongshan Ophthalmic Center, Sun Yat-Sen University, Guangzhou 510060, China; liyuting3@mail.sysu.edu.cn (Y.-T.L.); liangxiaoling@gzzoc.com (X.-L.L.); 2 School of Public Health, Sun Yat-Sen University, Guangzhou 510080, China; wangy856@mail2.sysu.edu.cn (Y.W.); huxj26@mail2.sysu.edu.cn (X.-J.H.); chenjh286@mail2.sysu.edu.cn (J.-H.C.); liyy379@mail2.sysu.edu.cn (Y.-Y.L.); zhongqy33@mail2.sysu.edu.cn (Q.-Y.Z.); chengh83@mail2.sysu.edu.cn (H.C.); 3 School of Public Health, LKS Faculty of Medicine, The University of Hong Kong, Hong Kong, China; bedru@hku.hk; 4 EDU, Digital Education Holdings Ltd., KKR-1320 Kalkara, Malta; jose.pantaleon@edu.edu.mt; 5 Green Templeton College, University of Oxford, Oxford OX2 6HG, UK; 6 JC School of Public Health and Primary Care, Faculty of Medicine, The Chinese University of Hong Kong, Hong Kong, China; 7 General Practice and Primary Care, Institute of Health and Wellbeing, University of Glasgow, Glasgow G12 9LX, UK

**Keywords:** systolic blood pressure, diabetic retinopathy, association, type 2 diabetes management, risk factor, healthcare

## Abstract

A common diabetes-related microvascular complication is diabetic retinopathy (DR), yet associations between blood pressure (BP) and risks for DR in diabetic patients with normal BP received inadequate attention. This may lead to ‘clinical inertia’ in early DR prevention. We aimed to assess whether the extent to which systolic BP levels were associated with DR in patients with type 2 diabetes (T2DM) and normal BP were similar to that in those with concurrent hypertension. Data were collected from patients with T2DM attending ophthalmic check-up with primary care referral (*n* = 2510). BP measurements, clinical laboratory tests, and dilated fundus examination were conducted according to gold standard of diagnosis and routine clinical procedure. Of all subjects, over 40% were normotensive and one fifth were clinically diagnosed with DR. Systolic BP levels increased across DR categories of escalated severity irrespective of the coexistence of hypertension. Ordinal logistic regression analysis showed that an increased systolic BP was independently and significantly associated with DR (adjusted odds ratio [aOR] = 1.020, *p* < 0.001 for hypertensives; aOR = 1.019, *p* = 0.018 for normotensives), after adjusting for diabetes duration, sex, lifestyles, and haemoglobin A1c levels. Regular monitoring of systolic BP should not be neglected in routine diabetes management even when BP falls within the normal range. (200 words)

## 1. Introduction

Globally, it is projected that over one in ten (10.4%) adults aged 20–79 years will be living with a diagnosis of diabetes in 2040 [1]. As the most common form of diabetes that shares concomitant risk factors with hypertension, type 2 diabetes mellitus (T2DM) poses enormous threats to health care with increased risks of both adverse cardiovascular and microvascular events [2]. A common microvascular complication is diabetic retinopathy (DR), which affects over one third of all diabetic patients [3,4]. DR progresses sequentially from mild non-proliferative DR which represents an asymptomatic early stage, to severe proliferative DR which represents the more advanced stage. As the leading cause of diabetes-related visual impairment and blindness, a worsening of DR is a major fear of most diabetic patients who may suffer increased distress and live with reduced function in daily life [5].

Although early detection of DR through regular screening with a comprehensive eye examination is key to preventing vision loss, the limited availability of healthcare resources in low- and middle-income countries (LMICs) may prevent T2DM patients from receiving appropriate DR examinations [6,7]. Global evidence suggests that longer duration of diabetes, suboptimal level of glycaemic control, and hypertension are strongly related to the onset and progression of DR [8,9,10,11]. Beneficial effects have been demonstrated in blood glucose control to prevent DR among patients with T2DM, while simultaneous blood pressure (BP) control is of equal importance [12,13,14].

Data synthesized from randomized, interventional studies conducted primarily in North America and Europe suggest that treatments with targets of systolic BP <140 mmHg reduces the onset, or decreases the progression of DR in patients with T2DM and established hypertension [15,16]. However, a knowledge gap exists regarding the association between BP levels and risks for DR in diabetic patients whose BP levels fall within the normal range. Such unawareness perceived by primary care clinicians, endocrinologists, and other healthcare professionals in early prevention of DR may result in ‘clinical inertia’ [17] of usual care with less attention devoted to routine BP monitoring in a sizable proportion of T2DM population who are normotensive.

From a preventive care perspective, the associations between systolic BP levels and DR in normotensive patients with T2DM is highly relevant in the context of the growing challenge of DR prevention across many countries where hypertension and T2DM are most common long-term conditions encountered in routine care. The main objective of the study was to assess whether the extent to which systolic BP levels were associated with the presence of DR in T2DM patients without hypertension, were similar to that in their counterparts with concurrent hypertension. The hypothesis that systolic BP levels were not associated with risk for DR in normotensive, T2DM patients was tested.

## 2. Materials and Methods

### 2.1. Study Design

This was a cross-sectional analysis of the baseline data from a cohort of adult Chinese patients diagnosed with T2DM attending ophthalmoscopic exams, who were referred from primary care facilities through a specialist–generalist working alliance consisting of 18 community health centres (CHCs) in Guangzhou, southern China. Overall, we aimed to invite approximately 5% of local adults diagnosed with T2DM, whose routine health care were covered by the CHCs in the community, for ophthalmic check-up in a specialised hospital. Based on the local population census and prevalence of T2DM in the adult Chinese population, an estimated half of invited primary care patients attended ophthalmic care between the study commencement in November 2017 and the baseline enrolment completion in December 2019.

### 2.2. Setting and Data Source

A dilated, comprehensive eye examination was provided as part of the baseline, ophthalmic check-up at a national-leading tertiary hospital specialised in ophthalmology. An interviewer-administered questionnaire assessment on routine lifestyle behaviours, a physical examination on anthropometric parameters and BP, and a clinical laboratory test for haemoglobin A1c (HbA1c) and lipid profiles were performed with a venous blood sample collected onsite. The blood test results were retrieved electronically from the centralised clinical laboratory in the hospital through a computerised health record system.

### 2.3. Participants

The study participants included both T2DM patients who had coexisting hypertension (*n* = 1495) and who were normotensive (*n* = 1015). Diabetes was diagnosed as fasting plasma glucose (FPG) ≥ 7.0 mmol/L, or 2-h plasma glucose ≥11.1 mmol/L during a 75g oral glucose tolerance test, or HbA1C ≥ 6.5% [18]. The presence of T2DM was determined by primary care physicians. The inclusion criteria of patients were: (1) aged between 30–80 years; (2) clinically diagnosed with T2DM; and (3) having routine primary care attendance in the context of national basic public health (BPH) service delivery [19]. Patients who had mental impairment, or who were not able to complete the full ophthalmoscopic exams were excluded from the study.

### 2.4. Study Variables and Measurements

Information on age, sex, education level, current cigarette smoking, and regular alcohol drinking were collected on the basis of patients’ self-report. The short form of the international physical activity questionnaire (IPAQ) was used, and a high level of physical activity achieved for the last seven days was determined according to the scoring protocol [20]. BP was measured in a seated position by routinely validated automatic sphygmomanometers. The arm with the higher BP values was used. The average of two BP readings, 1–2 min apart, was recorded. Hypertension was defined as systolic BP ≥140 mmHg and/or diastolic BP ≥90 mmHg on repeated clinical measurements, or by the presence of physician-prescribed anti-hypertensive medications for the purpose of BP lowering in the past 12 months [21]. Lipid profiles including plasma cholesterol and triglycerides were directly measured using an automated AutoAnalyzer (Cobs8000, Roche Diagnostics, Germany). HbA1c was measured by an automated, high performance liquid chromatography system (G8, Sysmex Corporation, Japan).

### 2.5. Fundus Examination and Grading of Diabetic Retinopathy

A comprehensive ophthalmic assessment including a fundus examination was performed by qualified ophthalmic specialists using slit-lamp biomicroscopy with dilated pupils and fundus photography adhering to clinical quality assurance standards. The presence of microvascular abnormalities including microaneurysms, retinal haemorrhages, hard exudates, cotton wool spots, venous dilation and beading, intraretinal microvascular abnormalities, and abnormal growth of new blood vessels were assessed. The grading of DR was determined based on the worst eye, using the International Classification of DR Scale [8]. No apparent DR was defined as having no microvascular abnormalities. Mild non-proliferative DR was defined as having microaneurysms only. Moderate non-proliferative DR was defined as more than just microaneurysms but less than severe non-proliferative DR. Severe non-proliferative DR was defined as having intraretinal haemorrhages (≥20 in each quadrant), or definite venous beading (in two quadrants), or intra-retinal microvascular abnormalities (in one quadrant). Proliferative DR was defined as the presence of severe non-proliferative DR with coexisting neovascularisation or vitreous/pre-retinal haemorrhage.

### 2.6. Statistical Analysis

Data entry was performed by two trained research assistants using EpiData 3.1 (The EpiData Association, Odense, Denmark) with double verification. Descriptive analysis was performed to explore the demographic, lifestyle, and health characteristics of study subjects by the presence of concurrent hypertension. Between-group differences were assessed by independent *t*-tests or chi-square tests, where appropriate. The mean systolic BP levels with 95% confidence interval (CI) were plotted against the grading of DR in T2DM patients with and without comorbid hypertension, respectively. The Spearman’s rank correlation test was performed to assess the strength of relationship between mean systolic BP levels and DR categories of escalated severity among T2DM patients. Ordinal logistic regression analysis was conducted to examine patient-level risk factors associated with categories of DR in order of increasing severity, i.e., from no apparent DR to proliferative DR. Independent variables that were statistically significant in the univariate model were entered into the multiple regression model. The proportional odds assumption was tested to ensure the robustness of the model. A binary logistic regression analysis with the presence of any stage of DR as the outcome variable was conducted as a sensitivity analysis. Data analyses were conducted using Stata 15.1 (StataCorp LLC, 4905 Lakeway Drive, College Station, TX, USA). A *p* value less than 0.05 was considered statistically significant.

### 2.7. Ethics Consideration

All participants provided written informed consent. Data anonymisation was done by removing all patient identifiers from the dataset prior to data analysis. Ethics approval was sought and granted by the Zhongshan Ophthalmic Center Medical Ethics Committee (Ref: 2017KYPJ094) at Sun Yat-Sen University in accordance with the Declaration of Helsinki 2013.

## 3. Results

### 3.1. Characteristics of Study Subjects

A total of 2510 patients who fulfilled the eligibility criteria were included in the analysis, among whom, over 40% were normotensive patients with T2DM. The mean age of overall subjects was 64.84 years (standard deviation [SD] 7.96), and slightly more than half (57.6% [1445/2510] were females. Nearly two thirds (64.3%) of patients completed senior secondary school education or above. Over one in ten (11.4%) subjects were active smokers, and a small proportion (7.5%) of patients reported alcohol drinking regularly. Nearly half (46.1%) of patients had a high level of physical activity. Diabetic patients who were normotensive tended to be younger (62.6 vs. 66.4 years; *p* < 0.001), more physically active (50.6% vs. 43.0%; *p* < 0.001), and had a higher likelihood of being current smokers (14.6% vs. 9.2%; *p* < 0.001) than their counterparts who had concurrent hypertension (Table 1).

Overall, the average duration of T2DM was 8.56 years (SD 7.10), and diabetic patients who were normotensive tended to have a shorter duration of diabetes (7.92 vs. 9.00 years; *p* < 0.001) than those with coexisting hypertension. The distributions of suboptimal blood glucose control were similar in both groups (58.1% vs. 57.9% for HbA1c ≥ 6.5%; *p* = 0.920) albeit a slightly higher level of HbA1c (7.10 vs. 6.97; *p* = 0.023) was observed among T2DM patients without hypertension. Of all patients, slightly over one fifth (21.9%) were clinically diagnosed with DR. The distributions of DR grading were not significantly different between the two groups. The stages of mild and moderate non-proliferative DR prevailed in both groups (Table 2).

### 3.2. Association between Systolic Blood Pressure and the Grading of Diabetic Retinopathy

**Figure 1:**
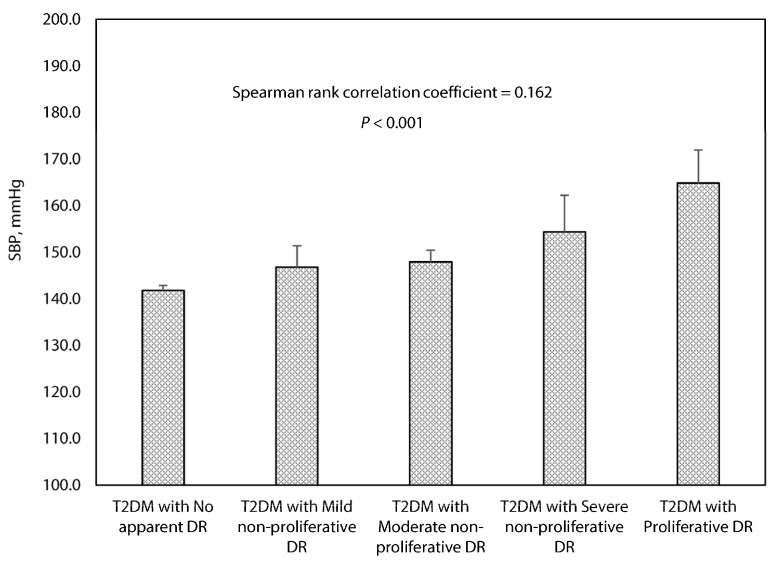
Association between systolic blood pressure and the grading of diabetic retinopathy in T2DM patients with concurrent hypertension. Error bars indicate 95% confidence interval. BP, blood pressure; DR, diabetic retinopathy. The stages of diabetic retinopathy are classified using the International Classification of DR Scale. Reference: International Diabetes Federation and the Fred Hollows Foundation. Diabetes eye health: a guide for health care professionals. Brussels, Belgium: International Diabetes Federation, 2015.

Among T2DM patients with comorbid hypertension, the mean systolic BP levels ranged from 141.8 mmHg (95%CI: 140.8–142.8 mmHg) for patients with no apparent DR to 164.8 mmHg (95%CI: 157.7–171.9 mmHg) for patients with proliferative DR, accompanying with a positive relationship (Spearman’s rank correlation test; *p* < 0.001) between systolic BP levels across DR categories of escalated severity (Figure 1).

**Figure 2:**
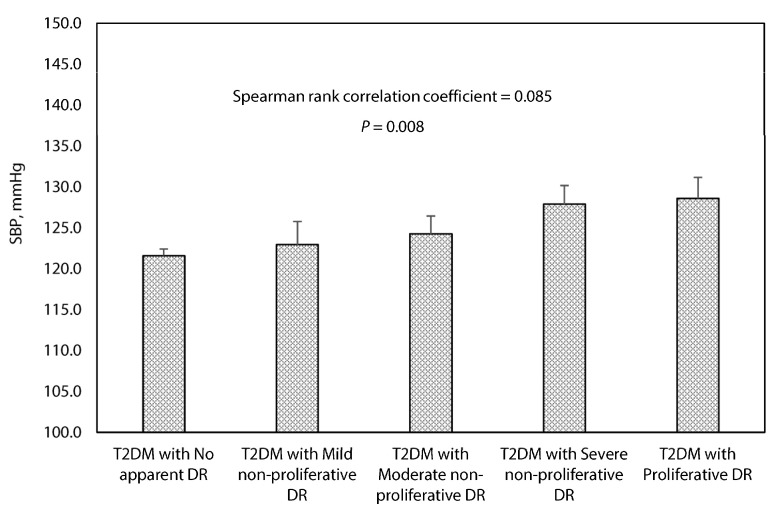
Association between systolic blood pressure and the grading of diabetic retinopathy in T2DM patients without hypertension. Error bars indicate 95% confidence interval. BP, blood pressure; DR, diabetic retinopathy. The stages of diabetic retinopathy are classified using the International Classification of DR Scale. Reference: International Diabetes Federation and the Fred Hollows Foundation. Diabetes eye health: a guide for health care professionals. Brussels, Belgium: International Diabetes Federation, 2015.

A similar pattern was observed among T2DM patients who were normotensive. The mean systolic BP levels ranged from 121.6 mmHg (95%CI: 120.8–122.4 mmHg) for patients with no apparent DR to 128.6 mmHg (95%CI: 126.0–131.2 mmHg) for patients with proliferative DR. A positive relationship (Spearman’s rank correlation test; *p* = 0.008) was also observed between systolic BP levels across DR categories in order of increasing severity (Figure 2).

### 3.3. Risk Factors Associated with Diabetic Retinopathy

Among T2DM patients with hypertension, univariate variables on duration of T2DM (*p* < 0.001), male sex (*p* < 0.001), regular drinking (*p* = 0.042), high physical activity (*p* = 0.017), BMI (*p* = 0.035), systolic BP levels (*p* < 0.001), and HbA1c levels (*p* < 0.001) were significantly associated with DR. The directions of significant associations remained unchanged in the adjusted model, although the strength of associations was slightly attenuated. After controlling for significant confounders including diabetes duration (aOR = 1.061, 95%CI: 1.042–1.081; *p* < 0.001), male sex (aOR = 1.733, 95%CI: 1.284–2.338; *p* < 0.001), high physical activity (aOR = 0.642, 95%CI: 0.478–0.861; *p* = 0.003), BMI (aOR = 0.950, 95%CI: 0.908-0.994; *p* = 0.028), and HbA1c levels (aOR = 1.527, 95%CI: 1.387–1.680; *p* < 0.001), systolic BP levels (aOR = 1.020, 95%CI: 1.012–1.029; *p* < 0.001) remained significantly associated with increased risk for DR in the ordinal logistic regression analysis (Table 3). Sensitivity analysis using a binary logistic regression model yielded similar results.

Similar findings were shown in both unadjusted and adjusted regression analysis for T2DM patients who were normotensive. After controlling for significant confounders including diabetes duration (aOR = 1.057, 95%CI: 1.032–1.082; *p* < 0.001) and HbA1c levels (aOR = 1.408, 95%CI: 1.275–1.555; *p* < 0.001), systolic BP levels (aOR = 1.019, 95%CI: 1.003–1.037; *p* = 0.018) remained significantly associated with increased risk for DR in the ordinal logistic regression analysis (Table 4). Sensitivity analysis using a binary logistic regression model yielded similar results.

## 4. Discussion

### 4.1. Main Findings

Our baseline data from a cohort of 2510 adult Chinese patients with T2DM, including both hypertensives and normotensives referred from primary care, demonstrated significant positive correlations between systolic BP levels and DR categories of escalated severity irrespective of the coexistence of hypertension. The systolic BP levels were significantly associated with the presence of DR, after adjusting for duration of T2DM, sex, lifestyle behaviours such as active smoking or regular drinking, and HbA1c levels in both hypertensive and normotensive patients with T2DM.

### 4.2. Relationship with Other Studies

When compared to a previous systematic review in which 26.9% [202 out of 751] of Chinese patients had the presence of any stage of DR [11], our study had a slightly lower proportion (21.9%) of patients with DR. This may be partly explained by the differences in methods used in ascertaining diabetes and DR status, and in distributions of patients with coexisting hypertension between our study and other study source. A diagnosis of T2DM based on patients’ self-report alone may have erroneously excluded those with undiagnosed diabetes from sample denominator, and may thus have overestimated the proportion of patients with DR when compared to that of patients in our study where a ‘gold standard’ blood test was used. Similar to previous studies in the UK [22] and multi-ethnic Asian population [23], we found that BMI levels were inversely associated with DR, and that increased physical activity was associated with less severe levels of DR, independent of the effects of HbA1c and BMI. Our logistic regression analysis also showed that longer duration of diabetes, elevated HbA1c and systolic BP levels were independently associated with the presence of DR. These were consistent with previous findings from the US and south Asian population [24,25], implying an international comparability of our study sample.

The association between systolic BP control and decreased progression, or reduced onset of DR were documented in earlier intervention studies that were mostly conducted among adult T2DM patients prescribed with antihypertensive medication treatments [15,16,26,27]. Although the debate on intensive vs. standard BP control targets in limiting the onset and progression of diabetic microvascular complications remains an area of controversy, the association between systolic BP and the development of DR was more apparent in those who had poorly controlled hypertension or those with a relatively longer follow-up period as shown in the UK Prospective Diabetes Study (UKPDS) and the Genetics of Diabetes Audit and Research in Tayside Scotland (GoDARTS) [27,28,29]. Similar findings of such association observed between systolic BP and DR in T2DM patients were also recently reported in both cross-sectional and retrospective cohort studies in the Asian population [30,31,32]. Nevertheless, the vast majority of T2DM patients recruited in existing studies had the presence of comorbid hypertension. This may affect the wider applicability of study findings to the normotensive group who have tended to be neglected. Although our study was not designed for establishing a threshold of systolic BP below which the risk of DR no longer decreased [33], our findings suggest that the extent to which systolic BP levels in T2DM patients without hypertension were associated with the presence of DR were similar to that in those with concurrent hypertension.

From a multimorbidity perspective, hypertension is approximately twice as common in patients with diabetes compared to those without it [33]. The association between elevated BP levels and poor microvascular outcomes such as DR is considered unequivocal and independent of other confounding risk factors in diabetic patients, as the evidence indicated an involvement of chronic inflammation and oxidative stress caused by hypertension [34]. However, suboptimal control of BP has been commonly seen in patients with diabetes partly as a result of ‘clinical inertia’ in routine practice [35,36]. This phenomenon even existed in clinical trials specifically designed for enhancing treatment adherence where more than half of T2DM patients had not-at-goal systolic BP levels [37]. The inertia in BP control may also play a role in the onset and progression of DR. A recent observational cohort study conducted among T2DM patients in southeast Asia reported an association between ‘clinical inertia’ and development of DR [17]. In our study, the observed associations between elevated systolic BP levels and increased risks for DR that were consistently shown among T2DM patients with and without concurrent hypertension are compatible with previous longitudinal findings, which highlight the importance of overcoming clinical inertia in routine BP monitoring in T2DM management. Moreover, we observed that T2DM patients without hypertension were more likely to be current smokers. We speculate that the level of motivation might play a role in those participants who smoked, as literature suggests that receiving a diagnosis of hypertension is more likely to provide additional impetus for cessation [38]. Hence, T2DM patients with normal BP might be less motivated to quit smoking in the absence of additional cardiovascular risk factors such as hypertension.

Compared to other studies, where 24 h-ambulatory BP monitoring was used in the stratification of different hypertensive phenotypes [39,40], the BP values used in our analysis were retrieved from clinical measurement conducted onsite at the ophthalmic visit. Hence the probable existence of the ‘white-coat hypertension’ was not specially accounted for. However, this is unlikely to distort the current study findings as the positive correlations observed between systolic BP levels across DR categories in order of increasing severity among hypertensives was similar in normotensives with T2DM. As such, our results also echo an earlier intervention in the US where the progression of DR was effectively reduced through telemedicine-mediated medication management and nurse-administered behavioural intervention for hypertension facilitated by home BP monitoring [41].

### 4.3. Implications for Research and Practice

A barrier to effective prevention of DR is that patients may not notice any obvious symptoms or changes to their vision during the early stage of DR until the condition has progressed to the point of significant vision loss. This shall require an individualised, shared-decision making approach that takes into account multifactorial aspects in blood glucose and BP control to reduce the risk for DR onset and progression. The landmark trial of Appropriate Blood Pressure Control in Diabetes (ABCD) reported beneficial effects of intensive BP control in slowing the progression of DR and lowering the incidence of stroke among patients with T2DM in the normotensive ABCD study over a 5-year interventional phase with antihypertensive therapy [42], although there remains no consensus towards a clear BP target [43]. Our results are in line with the recent AACE/ACE Consensus Statement [13], the ESC/EASD Guideline [44], and the ADA Standards of Medical Care in Diabetes [45] regarding the implications for sustained, optimal BP control in reducing the risk of diabetic microvascular complications. Notably, our finding on the association between systolic BP levels and the presence of DR is not necessarily indicative of any specific therapeutic threshold of systolic BP for the management of T2DM; but rather, reinforce the importance of regular monitoring of BP in all T2DM patients irrespective of the coexistence of hypertension.

To address the gaps in the evidence with respect to optimal targets for T2DM treatment, large-scale studies that span diverse patient samples are yet to be carried out in the context of population-wide health care triage and referral system with the assistance of electronic health record. This would enable novel insights from longitudinal investigations into the association between changes in clinical parameters at regular intervals and microvascular complications among T2DM patients who are free of other cardiovascular risk factors, and/or presented with various combinations of risk profiles and multimorbidity [46]. Meanwhile, joint efforts should be made with a proactive, multidisciplinary team approach [47] involving primary care generalists, endocrinologists, ophthalmic specialists, and healthcare professionals to enhance awareness of the sight-threatening risks in both hypertensive and normotensive patients with T2DM through early detection and treatment of diabetes-related complications. Moreover, we need research evidence from further longitudinal investigations using meaningful systolic BP intervals, e.g., 5–10 mmHg increases, to shed light on whether a cut-off point of systolic BP exists when clinical physicians should intervene, regardless of a diagnosis of hypertension. These efforts to be made in the next steps shall provide important guidance for clinical decisions.

### 4.4. Strengths and Weaknesses of the Study

We collected data from a relatively large sample of patients with medium-to-long duration of T2DM who were referred from primary care to ophthalmic check-up at a specialist hospital. Information collected covered aspects of patients’ demographics, lifestyles, disease history and outcome-oriented routine clinical parameters. All clinical measures including laboratory tests and fundus examination were performed according to gold standard of diagnosis and clinical check-up procedure with quality control. The study achieved a high level of data completeness, with only less than 3% of study subjects having ungradable images for DR due to small pupils, media opacities, or poor image quality.

However, our study has several limitations. Firstly, causal inferences cannot be simply drawn using a cross-sectional analytical approach. It is worth noting that the main purpose of the current analysis was not to assess the legacy effect of BP in the progression of DR or suggest any specific BP targets per se. Instead, we are more interested to explore the extent to which systolic BP levels in T2DM patients without hypertension were associated with the presence of DR, were similar to that in T2DM patients with concurrent hypertension. Secondly, there were several unmeasured confounders regarding process of care. This may include the use of medications, treatment adherence, prior service utilisation of eye checks, and experiences with primary care [48] where patients were referred, etc., which could potentially intervene the associations between systolic BP levels and DR. Nevertheless, the primary care settings in the study were of the same practice model and all adult patients with T2DM were offered routine health care and standard medical treatments in line with the national basic public health (BPH) service provision guideline for hypertension and type 2 diabetes management [19]. Moreover, all the dilated-pupil fundus examination procedures were performed centralised by a regularly trained team who followed standard clinical operating procedures. The impact of heterogeneity in the process of care on health outcomes can therefore be considered minimal. Thirdly, the presence of ‘white-coat hypertension’ may be unavoidable in the clinical setting. Ultimately, the current study design mainly targeted primary care patients under routine hypertension and T2DM management, which may affect the generalisability of our study findings to the general patient population of T2DM. However, it is reasonable to assume that those who are not regular service users of primary care, or walk-in patients without primary care referral, tend to have a poorer routine BP control, and hence the strength of associations between systolic BP and DR might be much stronger in those patient groups.

## 5. Conclusions

Our data suggested that systolic BP levels increased across the DR categories irrespective of the coexistence of hypertension. A higher systolic BP level was an independent, significant risk factor associated with the presence of DR, after adjusting for confounders in both hypertensive and normotensive patients with T2DM. Practicing clinicians, healthcare professionals, patients and care givers should not neglect the monitoring of systolic BP levels in diabetes management even when BP falls within the normal range.

## Figures and Tables

**Table 1:** Demographic and lifestyle characteristics of study participants.

	Total (*n* = 2510)	Hypertensives with T2DM (*n* = 1495)	Normotensives with T2DM (*n* = 1015)	*p*-Value
Age, years	64.84 (7.96)	66.36 (7.65)	62.61 (7.89)	<0.001
Age (groups)				
<60 years	616 (24.5%)	274 (18.3%)	342 (33.7%)	<0.001
60–69 years	1230 (49.0%)	728 (48.7%)	502 (49.5%)	
≥70 years	664 (26.5%)	493 (33.0%)	171 (16.8%)	
Sex				
Women	1445 (57.6%)	888 (59.4%)	557 (54.9%)	0.024
Men	1065 (42.4%)	607 (40.6%)	458 (45.1%)	
Education level				
≥Senior secondary	1614 (64.3%)	925 (61.9%)	682 (67.2%)	0.066
Others	896 (35.7%)	570 (38.1%)	333 (32.8%)	
Current smoking				
Yes	286 (11.4%)	138 (9.2%)	148 (14.6%)	<0.001
No	2224 (88.6%)	1357 (90.8%)	867 (85.4%)	
Regular drinking				
Yes	189 (7.5%)	100 (6.7%)	89 (8.8%)	0.053
No	2321 (92.5%)	1395 (93.3%)	926 (91.2%)	
Levels of physical activity				
Low-to-moderate	1353 (53.9%)	852 (57.0%)	501 (49.4%)	<0.001
High	1157 (46.1%)	643 (43.0%)	514 (50.6%)	

T2DM, type 2 diabetes mellitus. Data are presented as mean (standard deviation) or *n* (%), where appropriate. Column percentages were derived from the total number in the corresponding column.

**Table 2:** Diabetes duration, haemoglobin A1c, blood pressure, and diabetic retinopathy of study participants.

	Total (*n* = 2510)	Hypertensives with T2DM (*n* = 1495)	Normotensives with T2DM (*n* = 1015)	*p*-Value
Duration of diabetes, years	8.56 (7.10)	9.00 (7.22)	7.92 (6.87)	<0.001
Duration of diabetes (groups)				
<4 years	768 (30.6%)	433 (29.0%)	335 (33.0%)	0.004
4–11 years	1023 (40.8%)	598 (40.0%)	425 (41.9%)	
≥12 years	719 (28.6%)	464 (31.0%)	255 (25.1%)	
HbA1c, %	7.02 (1.43)	6.97 (1.38)	7.10 (1.51)	0.023
HbA1c (groups)				
<6.5%	1054 (42.0%)	629 (42.1%)	425 (41.9%)	0.920
≥6.5%	1456 (58.0%)	866 (57.9%)	590 (58.1%)	
Systolic BP, mmHg	134.82 (18.74)	143.39 (17.86)	122.14 (11.40)	<0.001
Diastolic BP, mmHg	70.45 (10.47)	72.89 (10.97)	66.80 (8.44)	<0.001
Presence of DR				
No	1960 (78.1%)	1155 (77.3%)	805 (79.3%)	0.222
Yes	550 (21.9%)	340 (22.7%)	210 (20.7%)	
Grading of DR ^1^				
Mild NPDR	119 (22.3%)	61 (18.4%)	58 (28.6%)	0.081
Moderate NPDR	342 (64.0%)	222 (67.1%)	120 (59.1%)	
Severe NPDR	47 (8.8%)	30 (9.1%)	17 (8.4%)	
Proliferative DR	26 (4.9%)	18 (5.4%)	8 (3.9%)	

^1^ Patients with ungradable images for diabetic retinopathy due to small pupils, media opacities, or poor image quality were excluded (*n* = 16). T2DM, type 2 diabetes mellitus; BP, blood pressure; HbA1c, haemoglobin A1c; DR, diabetic retinopathy; NPDR, non-proliferative diabetic retinopathy. Data are presented as mean (standard deviation) or *n* (%), where appropriate. Column percentages were derived from the total number in the corresponding column.

**Table 3:** Risk factors associated with diabetic retinopathy in hypertensive patients with T2DM.

Variables	Crude Model		Adjusted Model	
	cOR (95%CI)	*p*-Value	aOR (95%CI)	*p*-Value
Duration, years	1.077 (1.058–1.096)	<0.001	1.061 (1.042–1.081)	<0.001
Male sex	1.688 (1.294–2.202)	<0.001	1.733 (1.284–2.338)	<0.001
Regular drinking	1.605 (1.016–2.535)	0.042	1.146 (0.691–1.901)	0.596
High physical activity	0.717 (0.547–0.942)	0.017	0.642 (0.478–0.861)	0.003
BMI	0.956 (0.917–0.997)	0.035	0.950 (0.908–0.994)	0.028
Systolic BP	1.025 (1.017–1.033)	<0.001	1.020 (1.012–1.029)	<0.001
HbA1c	1.631 (1.488–1.787)	<0.001	1.527 (1.387–1.680)	<0.001
Age, years	0.989 (0.972–1.006)	0.207	-	-
High education level	0.840 (0.635–1.110)	0.219	-	-
Current smoking	1.130 (0.730–1.748)	0.583	-	-
Diastolic BP	1.004 (0.992–1.016)	0.517	-	-
Triglyceride	0.998 (0.926–1.075)	0.955	-	-
Total cholesterol	0.967 (0.853–1.097)	0.605	-	-
LDL cholesterol	0.962 (0.836–1.107)	0.589	-	-
HDL cholesterol	0.982 (0.700–1.377)	0.914	-	-

cOR, crude odds ratio; aOR, adjusted odds ratio; CI, confidence interval; BP, blood pressure; T2DM, type 2 diabetes mellitus; LDL, low-density lipoprotein; HDL, high-density lipoprotein; BMI, body mass index; HbA1c, haemoglobin A1c. Independent variables on male sex, high education level, regular drinking, current smoking, and high physical activity were dichotomous, while the other variables were continuous. Independent variables that were significantly associated with the dependent variable (Y = categories of diabetic retinopathy in order of increasing severity, i.e., from no apparent DR to proliferative DR) in the crude model were entered into the adjusted model.

**Table 4:** Risk factors associated with diabetic retinopathy in normotensive patients with T2DM.

Variables	Crude Model		Adjusted Model	
	cOR (95%CI)	*p*-Value	aOR (95%CI)	*p*-Value
Duration, years	1.066 (1.043–1.091)	<0.001	1.057 (1.032–1.082)	<0.001
Current smoking	1.572 (1.015–2.435)	0.043	1.358 (0.857–2.150)	0.192
Systolic BP	1.024 (1.008–1.040)	0.004	1.019 (1.003–1.037)	0.018
HbA1c	1.465 (1.329–1.614)	<0.001	1.408 (1.275–1.555)	<0.001
Male sex	1.241 (0.883–1.742)	0.213	-	-
Age, years	0.983 (0.962–1.004)	0.120	-	-
High education level	0.819 (0.582–1.153)	0.252	-	-
Regular drinking	0.843 (0.447–1.589)	0.597	-	-
High physical activity	0.777 (0.553–1.092)	0.146	-	-
BMI	0.964 (0.913–1.018)	0.188	-	-
Diastolic BP	0.990 (0.971–1.010)	0.344	-	-
Triglyceride	0.963 (0.858–1.082)	0.530	-	-
Total cholesterol	0.924 (0.785–1.088)	0.344	-	-
LDL cholesterol	0.991 (0.827–1.187)	0.922	-	-
HDL cholesterol	0.728 (0.469–1.130)	0.157	-	-

cOR, crude odds ratio; aOR, adjusted odds ratio; CI, confidence interval; BP, blood pressure; T2DM, type 2 diabetes mellitus; LDL, low-density lipoprotein; HDL, high-density lipoprotein; BMI, body mass index; HbA1c, haemoglobin A1c. Independent variables on male sex, high education level, regular drinking, current smoking, and high physical activity were dichotomous, while the other variables were continuous. Independent variables that were significantly associated with the dependent variable (Y = categories of diabetic retinopathy in order of increasing severity, i.e., from no apparent DR to proliferative DR) in the crude model were entered into the adjusted model.

## Data Availability

The data presented in this study are available on reasonable request from the corresponding author.

## References

[1] Ogurtsova K., da Rocha Fernandes J.D., Huang Y., Linnenkamp U., Guariguata L., Cho N.H., Cavan D., Shaw J.E., Makaroff L.E. (2017). IDF Diabetes Atlas: Global estimates for the prevalence of diabetes for 2015 and 2040. Diabetes Res. Clin. Pract..

[2] Beckman J.A., Creager M.A. (2016). Vascular complications of diabetes. Circ Res..

[3] Solomon S.D., Chew E., Duh E.J., Sobrin L., Sun J.K., VanderBeek B.L., Wykoff C.C., Gardner T.W. (2017). Diabetic Retinopathy: A Position Statement by the American Diabetes Association. Diabetes Care.

[4] Harding J.L., Pavkov M.E., Magliano D.J., Shaw J.E., Gregg E.W. (2019). Global trends in diabetes complications: A review of current evidence. Diabetologia.

[5] Simó-Servat O., Hernández C., Simó R. (2019). Diabetic Retinopathy in the Context of Patients with Diabetes. Ophthalmic Res..

[6] Gale M.J., Scruggs B.A., Flaxel C.J. (2021). Diabetic eye disease: A review of screening and management recommendations. Clin. Exp. Ophthalmol..

[7] Ting D.S.W., Cheung G.C.M., Wong T.Y. (2016). Diabetic retinopathy: Global prevalence, major risk factors, screening practices and public health challenges: A review. Clin. Exp. Ophthalmol..

[8] International Diabetes Federation and The Fred Hollows Foundation (2015). Diabetes Eye Health: A Guide for Health Care Professionals.

[9] Lanzetta P., Sarao V., Scanlon P.H., Barratt J., Porta M., Bandello F., Loewenstein A. (2020). Fundamental principles of an effective diabetic retinopathy screening program. Acta Diabetol..

[10] Rodriguez-Poncelas A., Miravet-Jiménez S., Casellas A., Barrot-De La Puente J.F., Franch-Nadal J., López-Simarro F., Mata-Cases M., Mundet-Tudurí X. (2015). Prevalence of diabetic retinopathy in individuals with type 2 diabetes who had recorded diabetic retinopathy from retinal photographs in Catalonia (Spain). Br. J. Ophthalmol..

[11] Yau J.W., Rogers S.L., Kawasaki R., Lamoureux E.L., Kowalski J.W., Bek T., Chen S.-J., Dekker J.M., Fletcher A., Grauslund J. (2012). Global prevalence and major risk factors of diabetic retinopathy. Diabetes Care.

[12] McGill J.B. (2009). Improving microvascular outcomes in patients with diabetes through management of hypertension. Postgrad Med..

[13] Garber A.J., Handelsman Y., Grunberger G., Einhorn D., Abrahamson M.J., Barzilay J.I., Blonde L., Bloomgarden Z.T., Bush M.A., American Association of Clinical Endocrinologists (2020). Consensus Statement by the American Association of Clinical Endocrinologists and American College of Endocrinology on the Comprehensive Type 2 Diabetes Management Algorithm-2020 Executive Summary. Endocr. Pract..

[14] Valencia W.M., Florez H. (2017). How to prevent the microvascular complications of type 2 diabetes beyond glucose control. BMJ.

[15] Do D.V., Wang X., Vedula S.S., Marrone M., Sleilati G., Hawkins B.S., Frank R.N. (2015). Blood pressure control for diabetic retinopathy. Cochrane Database Syst. Rev..

[16] Emdin C.A., Rahimi K., Neal B., Callender T., Perkovic V., Patel A. (2015). Blood pressure lowering in type 2 diabetes: A systematic review and meta-analysis. JAMA.

[17] Osataphan S., Chalermchai T., Ngaosuwan K. (2016). Clinical inertia causing new or progression of diabetic retinopathy in type 2 diabetes: A retrospective cohort study. J. Diabetes.

[18] Weng J., Ji L., Jia W., Lu J., Zhou Z., Zou D., Zhu D., Chen L., Chen L., Guo L. (2016). Standards of care for type 2 diabetes in China. Diabetes Metab. Res. Rev..

[19] Department of Primary Health, National Health Commission, PRC 2017, No.13 Document. Standards for National Basic Public Health Services (3rd edition). http://www.nhc.gov.cn/jws/s3578/201703/d20c37e23e1f4c7db7b8e25f34473e1b.shtml.

[20] IPAQ Research Committee (2005). Guidelines for Data Processing and Analysis of the International Physical Activity Questionnaire (IPAQ)-Short and Long Forms. http://www.ipaq.ki.se/.

[21] Writing Group of the 2018 Chinese Guidelines for the Management of Hypertension (2019). 2018 Chinese guidelines for the management of hypertension. Chin. J. Cardiovasc. Med..

[22] Praidou A., Harris M., Niakas D., Labiris G. (2017). Physical activity and its correlation to diabetic retinopathy. J. Diabetes Complicat..

[23] Rooney D., Lye W.K., Tan G., Lamoureux E.L., Ikram M.K., Cheng C.Y., Kumari N., Zheng Y.F., Mitchell P., Wang J.J. (2015). Body mass index and retinopathy in Asian populations with diabetes mellitus. Acta Diabetol..

[24] Zhang X., Saaddine J.B., Chou C.-F., Cotch M.F., Cheng Y.J., Geiss L.S., Gregg E.W., Albright A.L., Klein B.E.K., Klein R. (2010). Prevalence of Diabetic Retinopathy in the United States, 2005–2008. JAMA.

[25] Raman R., Gupta A., Krishna S., Kulothungan V., Sharma T. (2012). Prevalence and risk factors for diabetic microvascular complications in newly diagnosed type II diabetes mellitus. Sankara Nethralaya Diabetic Retinopathy Epidemiology and Molecular Genetic Study (SN-DREAMS, report 27). J. Diabetes Complicat..

[26] Sjølie A.K., Klein R., Porta M., Orchard T., Fuller J., Parving H.H., Bilous R., Chaturvedi N. (2008). Effect of candesartan on progression and regression of retinopathy in type 2 diabetes (DIRECT-Protect 2): A randomised placebo-controlled trial. Lancet.

[27] Adler A.I., Stratton I.M., Neil H.A., Yudkin J.S., Matthews D.R., Cull C.A., Holman R.R. (2000). Association of systolic blood pressure with macrovascular and microvascular complications of type 2 diabetes (UKPDS 36): Prospective observational study. BMJ.

[28] Liu Y., Wang M., Morris A.D., Doney A.S., Leese G.P., Pearson E.R., Palmer C.N. (2013). Glycemic exposure and blood pressure influencing progression and remission of diabetic retinopathy: A longitudinal cohort study in GoDARTS. Diabetes Care.

[29] Porta M., Taulaigo A.V. (2014). The changing role of the endocrinologist in the care of patients with diabetic retinopathy. Endocrine.

[30] Foo V., Quah J., Cheung G., Tan N.C., Zar K.L.M., Chan C.M., Lamoureux E., Yin W.T., Tan G., Sabanayagam C. (2016). HbA1c, systolic blood pressure variability and diabetic retinopathy in Asian type 2 diabetics. J. Diabetes.

[31] Liu L., Quang N.D., Banu R., Kumar H., Tham Y.C., Cheng C.Y. (2020). Hypertension, blood pressure control and diabetic retinopathy in a large population-based study. PLoS ONE.

[32] Takao T., Matsuyama Y., Suka M., Yanagisawa H., Kikuchi M., Kawazu S. (2014). Time-to-effect relationships between systolic blood pressure and the risks of nephropathy and retinopathy in patients with type 2 diabetes. J. Diabetes Complicat..

[33] Williams M.E. (2011). The goal of blood pressure control for prevention of early diabetic microvascular complications. Curr. Diab. Rep..

[34] Duarte D.A., Silva K.C., Rosales M.A., Lopes de Faria J.B., Lopes de Faria J.M. (2013). The concomitance of hypertension and diabetes exacerbating retinopathy: The role of inflammation and oxidative stress. Curr. Clin. Pharmacol..

[35] Phillips L.S., Branch W.T., Cook C.B., Doyle J.P., El-Kebbi I.M., Gallina D.L., Miller C.D., Ziemer D.C., Barnes C.S. (2001). Clinical Inertia. Ann. Intern. Med..

[36] Berlowitz D.R., Ash A.S., Hickey E.C., Glickman M., Friedman R., Kader B. (2003). Hypertension management in patients with diabetes: The need for more aggressive therapy. Diabetes Care.

[37] Barton A.B., Okorodudu D.E., Bosworth H.B., Crowley M.J. (2018). Clinical Inertia in a Randomized Trial of Telemedicine-Based Chronic Disease Management: Lessons Learned. Telemed. J. E Health.

[38] Shahab L., Mindell J., Poulter N.R., West R. (2010). Hypertension and its identification among current, past and never smokers in an English population sample. Eur. J. Cardiovasc Prev. Rehabil..

[39] Leiria L.F., Severo M.D., Ledur P.S., Becker A.D., Aguiar F.M., Massierer D., Freitas V.C., Schaan B.D., Gus M. (2015). White coat effect and masked uncontrolled hypertension in treated hypertensive-diabetic patients: Prevalence and target organ damage. J. Diabetes..

[40] Head G.A., Shaw J.E., Dunstan D.W., Owen N., Magliano D.J., Chadban S., Zimmet P. (2019). Hypertension, white-coat hypertension and masked hypertension in Australia: Findings from the Australian Diabetes, Obesity, and Lifestyle Study 3. J. Hypertens..

[41] Muir K.W., Grubber J., Mruthyunjaya P., McCant F., Bosworth H.B. (2013). Progression of diabetic retinopathy in the hypertension intervention nurse telemedicine study. JAMA Ophthalmol..

[42] Schrier R.W., Estacio R.O., Mehler P.S., Hiatt W.R. (2007). Appropriate blood pressure control in hypertensive and normotensive type 2 diabetes mellitus: A summary of the ABCD trial. Nat. Clin. Pract. Nephrol..

[43] Vijan S. (2014). Diabetes: Treating hypertension. BMJ Clin. Evid..

[44] Cosentino F., Grant P.J., Aboyans V., Bailey C.J., Ceriello A., Delgado V., Federici M., Filippatos G., Grobbee D.E., Hansen T.B. (2020). 2019 ESC Guidelines on diabetes, pre-diabetes, and cardiovascular diseases developed in collaboration with the EASD. Eur. Heart J..

[45] American Diabetes Association (2021). 10. Cardiovascular Disease and Risk Management: Standards of Medical Care in Diabetes-2021. Diabetes Care.

[46] Wong M.C.S., Wang H.H.X., Cheung C.S.K., Tong E.L.H., Sek A.C.H., Cheung N.T., Yan B.P.Y., Yu C.M., Griffiths S.M., Coats A.J.S. (2014). Factors associated with multimorbidity and its link with poor blood pressure control among 223,286 hypertensive patients. Int. J. Cardiol..

[47] Wang H.H.X. (2020). Taking a multidisciplinary team approach to better healthcare outcomes for society. Hong Kong Med. J..

[48] Wang H.H.X., Wong S.Y.S., Wong M.C.S., Wei X.L., Wang J.J., Li D.K.T., Tang J.L., Gao G.Y., Griffiths S.M. (2013). Patients’ experiences in different models of community health centers in southern China. Ann. Fam. Med..

